# The Effect of the Orientation towards Analyte Flow on Electrochemical Sensor Performance and Current Fluctuations

**DOI:** 10.3390/s20041038

**Published:** 2020-02-14

**Authors:** Petr Sedlák, Petr Kuberský

**Affiliations:** 1 Faculty of Electrical Engineering and Communications, Brno University of Technology, Technická 10, 616 00 Brno, Czech Republic; 2 Faculty of Electrical Engineering, Regional Innovation Centre for Electric Engineering, University of West Bohemia, Univerzitni 8, 301 00 Plzen, Czech Republic; kubersky@ket.zcu.cz

**Keywords:** analyte flow, current fluctuations, amperometric sensor, signal-to-noise ratio

## Abstract

Analyte flow influences the performance of every gas sensor; thus, most of these sensors usually contain a diffusion barrier (layer, cover, inlet) that can prevent the negative impact of a sudden change of direction and/or the rate of analyte flow, as well as various unwanted impacts from the surrounding environment. However, several measurement techniques use the modulation of the flow rate to enhance sensor properties or to extract more information about the chemical processes that occur on a sensitive layer or a working electrode. The paper deals with the experimental study on how the analyte flow rate and the orientation of the electrochemical sensor towards the analyte flow direction influence sensor performance and current fluctuations. Experiments were carried out on a semi-planar, three-electrode topology that enabled a direct exposure of the working (sensing) electrode to the analyte without any artificial diffusion barrier. The sensor was tested within the flow rate range of 0.1–1 L/min and the orientation of the sensor towards the analyte flow direction was gradually set to the four angles 0°, 45°, 90° and 270° in the middle of the test chamber, while the sensor was also investigated in the standard position at the bottom of the chamber.

## 1. Introduction

Chemical gas sensors represent low-cost, easy-to-use devices with a high miniaturization potential to detect chemical substances dispersed in an environment for a wide spectrum of applications [1,2]. The sensor mechanisms and their properties are tightly bound with electric charge transport at/in active electrochemical interfaces [3], and are deliberately influenced by changing working conditions or unintentionally by the outside environment. It should be noted that several measurement techniques use modulation of the flow rate [4,5,6,7], the temperature [8,9,10,11,12] or even the light [13,14,15] to extract more information about the chemical processes or to enhance sensor properties, since the electrochemical sensor (such as a conductometric or amperometric one) could exhibit cross-interference, i.e., non-selectivity, for chemically similar molecules [1,16].

The analyte flow rate influences the performance of every gas sensor; thus, most of these sensors usually contain a diffusion barrier (layer, cover or inlet) that may prevent the negative impact of a sudden change of direction and/or the rate of analyte flow, as well as various unwanted impacts from the surrounding environment [17]. Several authors described the effect of the flow rate on the signal and sensor properties of gas sensors [18,19,20,21]. Our previous paper [21] demonstrated on a fully-printed amperometric gas sensor that the direct current (DC) through the sensor exhibits changes within the range of one order for a non-zero flow rate, while the spectral density of current fluctuations significantly changes its shape and the level of current fluctuations in the power spectrum for the orders as the flow rate increases at a constant concentration of the detected gas. Furthermore, the signal-to-noise ratio of response was almost invariant to gas concentration and decreased with flow rate increase. These results indicate the importance of current fluctuation measurements, making it possible to extract new and valuable information (parameters) resulting in a fluctuation-enhanced detection technique. 

This paper deals with the experimental study of how the analyte flow rate and the orientation of the sensor, i.e., the surface area of the working electrode (WE) towards the analyte flow direction, impact overall sensor performance and current fluctuations. The first set of experiments was aimed at a comparison of selected sensor parameters (sensitivity, response/recovery time, limit of detection and repeatability) dependent on both the size of the WE surface area and the analyte flow rate. Within the second set of experiments, attention was paid to the effect of the mutual angle between the analyte flow direction and the WE surface area on sensor parameters, where the orientation of the sensor towards the analyte flow was gradually set to the four angles 0°, 45°, 90° and 270° in the middle of the test chamber. The third set of experiments was aimed at the study of current fluctuations depending on sensor rotation for the aforementioned angles between the analyte flow direction and the surface area of the working electrode. Two dependences were studied separately for each angle: (i) the effect of analyte concentration at a particular flow rate, (ii) the effect of analyte flow rate at a particular concentration.

## 2. Materials and Methods

### 2.1. Sensor Preparation

**Figure 1:**
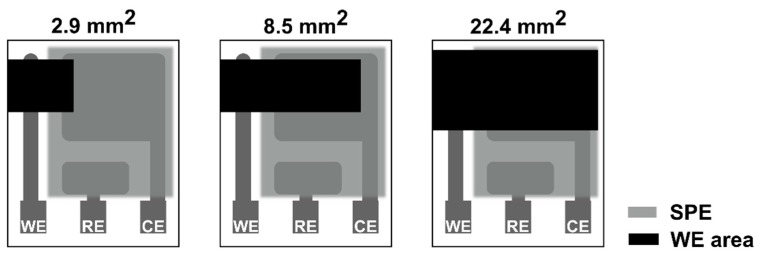
Sensor topology with three different working electrode areas.

All experiments were carried out on a well-established, three−electrode sensor platform (see Figure 1) based on a ceramic substrate with a platinum counter (CE) and a platinum pseudoreference electrode (RE). A solid polymer electrolyte (SPE) contained 1-ethyl-3-methylimidazolium bis(trifluoromethylsulfonyl)imide [C_2_mim][NTf_2_] ionic liquid, poly(vinylidene fluoride) (PVDF) and 1-methyl-2-pyrrolidone (NMP). More information about the sensor platform and a detailed description of SPE preparation can be found in [22,23,24] and [25,26], respectively. A carbon working electrode was prepared by spray coating a mixture consisting of 300 mg glassy carbon spherical powder (2−12 µm, Sigma−Aldrich, Germany) and 1 mL of ethanol. Three different working electrode areas were prepared with regard to the overall dimension of the basic ceramic substrate. The ratio of geometric areas between WE and CE were approximately 1/5,1/2 and 1 for the WE surface area 2.9, 8.5 and 22.4 mm^2^, respectively.

### 2.2. Experimental Setup

The apparatus for sensor testing consisted of PC-controlled mass flow controllers (SmartTrak100, Sierra Instruments, Monterey, USA-California), gas cylinders with synthetic air and a reference calibration mixture (100 ppm NO_2_ balanced in nitrogen), a test chamber and readout electronics. For further description of the apparatus, the authors refer to a previous study [21]. For the first set of experiments, when the tested sensors were placed onto the test chamber bottom, gold spring probes were used to contact all three electrodes. For the second and third set of experiments, the tested sensor was contacted via silver wires that allowed the rotation of the sensor. A programmable analog front-end (AFE) potentiostat LMP91000 (Texas Instrument, Dallas, USA) was used as readout electronics for all the measurements except the measurement of current fluctuation. Bias voltage between RE and WE was set to 0.5 V for all the experiments and the sensor response current was recorded every other second. 

The measurement setup for evaluation of current fluctuation were based on our own battery-fed device, which implements the potentiostat circuit in a grounded-WE configuration [27] and a low-noise transimpedance amplifier with separated alternating current (AC) and DC outputs. The sensor under test is connected to the potentiostat circuit. The AC voltage output was led to an amplifier with highly selective filters AM22 (3S Sedlak, s.r.o., Brno, Czech Republic), and was obtained by a 12-bit AD convertor HS5 (TiePie engineering, Sneek, Netherlands) as well as DC voltage output. To eliminate the external disturbance from the environment, the test chamber with the sensor and our device is located in a faraday cage and all devices of the measurement chain were powered from batteries.

## 3. Results and Discussion

**Figure 2:**
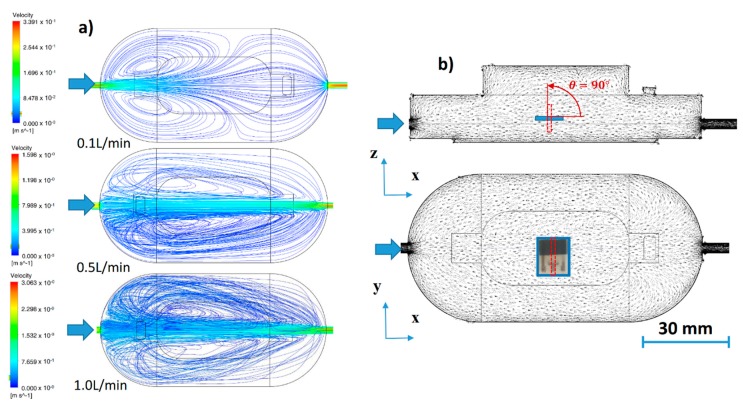
(**a**) Computational fluid dynamics (CFD) simulations of airflow in the test chamber at flow rates 0.1 L/min, 0.5 L/min and 1.0 L/min, (**b**) sensor position and orientation towards the flow at angle 0° and 90° in the gas chamber.

To illustrate airflow behavior in the test chamber at various flow rates, simplified preliminary simulations were carried out without the consideration of a sensor, contact needles, etc. Figure 2a shows computational fluid dynamics (CFD) simulations [28] of airflow in the gas chamber at the flow rates of 0.1 L/min, 0.5 L/min and 1.0 L/min. The computation was carried out in the software environment ANSYS 17.0 for the chamber without any sensor or contact needles. The CFD simulations show that vortices are formed for all rates, thus, the flow in the chamber is turbulent [29]. At 0.1 L/min, the flow along the longitudinal mid-plane exhibit minimal influence from vortices, while for higher flow rates the degree of turbulence grows. Two significant vortices are formed symmetrically to the longitudinal plane of the chamber, and change their positions from the left to the right as the flow rate increases. One of these two vortices is supposed to significantly contribute to the signal response of a sensor, since it directly influences the flow around the sensor. Figure 2b illustrates the position of the sensor during the measurements in the air as well as streamlines at the flow rate of 1 L/min. 

**Figure 3:**
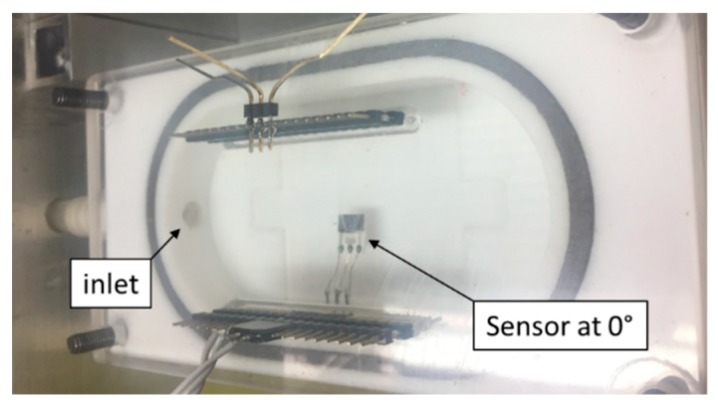
Sensor position in the gas chamber at angle 0°.

The orientation of the sensor towards the analyte flow was set gradually to the four angles of 0°, 45°, 90° and 270°, while the sensor was also investigated in the standard position at the bottom of the gas chamber [21]. According to simulations, the analyte flow from inlet seems to impact directly WE electrode of the sensor at angles 0°, 45° and 90°. Detailed simulations of the fluidic conditions in the vicinity of the sensor were not carried out due to their high complexity. Figure 3 shows the location and rotation of the sensor for the 0° angle.

### 3.1. Effect of the Working Electrode (WE) Surafce Area and Analyte Flow Rate on Sensor Parameters

**Figure 4:**
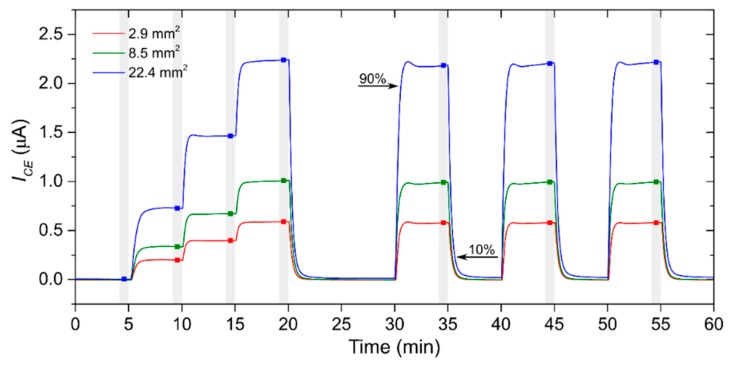
Sensor response to the test cycle for samples with different working electrode (WE) surface areas (conditions: 298 K, 40%RH (relative humidity), analyte flow rate 0.5 L/min).

Within the first set of experiments, we tested sensors with three different working electrode areas (2.9, 8.5 and 22.4 mm^2^) and compared their basic sensor parameters (sensitivity, response/recovery time, limit of detection, repeatability) in order to determine the influence of a different working electrode area on sensor parameters. Each sensor was exposed to the same test profile that consisted of a stepwise increase in nitrogen dioxide concentration from 0 to 3 ppm (1 step equaled 1 ppm NO_2_) with subsequent three consecutive exposures to the same concentration of 3 ppm NO_2_. We applied the same test profile with four different total flow rates of analyte (0.1, 0.5, 0.8 and 1 L/min) in order to observe the impact of the flow rate level on sensor parameters for each size of the working electrode area. Relative humidity and temperature were constant within all experiments (298 K and 40%RH unless otherwise stated) and each of the sensors tested was placed on the same position in the test chamber to be exposed to the most identical conditions. Figure 4 shows typical sensor responses to the test cycle. Sensitivity was determined as the slope of a calibration curve. For the construction of the calibration curve, an average value of sensor current was determined for each concentration. Average values (illustrated by red, green and blue squares in Figure 4) were calculated from the last minute of each concentration level (highlighted by the gray zone), where sensor responses were stable. Response/recovery time (T_90_/T_10_) was calculated as the time period necessary to achieve 90% or 10% of the steady state current upon a step increase/decrease in NO_2_ concentration. Further sensor parameters, the limit of detection (LOD) and repeatability, will be discussed in the following text.

**Figure 5:**
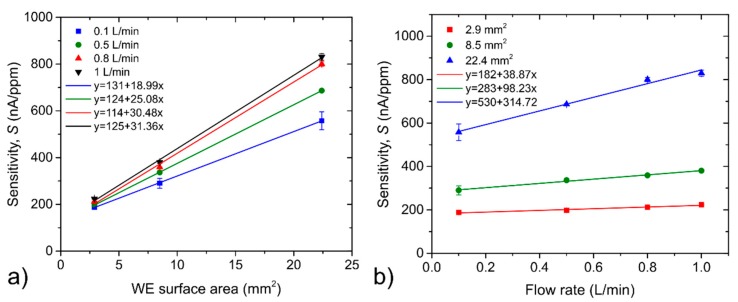
(**a**) Sensitivity dependence on the WE surface area; (**b**) sensitivity dependence on the analyte flow rate. The error bars in Figure 5 represent the standard error of the slope of the calibration curve (sensitivity) that was constructed by linear regression.

Figure 5 shows sensitivity dependence on the WE surface area for different analyte flow rates (Figure 5a) and on analyte flow rates for different WE surface areas (Figure 5b). Sensor sensitivity exhibited a linearly growing trend with the increase of both the WE surface area and the analyte flow rate. The increase of sensitivity per unit surface area was 19, 25.1, 30.5 and 31.4 nA/ppm for analyte flow rates of 0.1, 0.5, 0.8 and 1 L/min, respectively. The higher the flow rate, the higher the change of sensitivity per unit surface area. The increase of 100 mL/min of analyte flow rate caused a sensitivity increase of 3.9, 9.8 and 31.5 nA/ppm for the WE surface area of 2.9, 8.5 and 22.4 mm^2^, respectively. The larger the WE surface area, the higher change of sensitivity per unit analyte flow rate.

**Figure 6:**
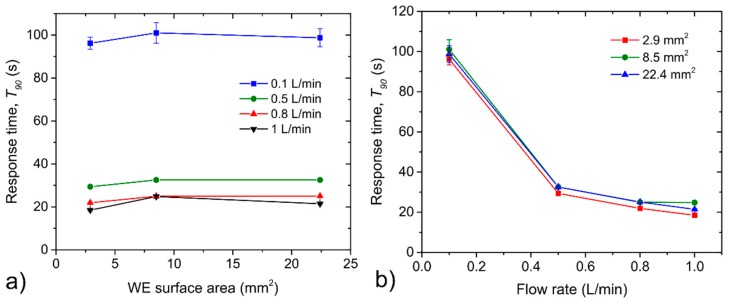
(**a**) Response time dependence on the WE surface area, (**b**) the response time dependence on the analyte flow rate. The error bars represent standard error of the mean that was calculated from three consecutive exposures to the same concentration.

**Figure 7:**
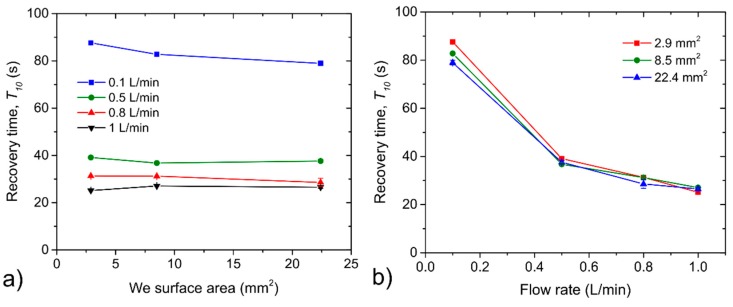
(**a**) Recovery time dependence on the WE surface area, (**b**) recovery time dependence on the analyte flow rate. The error bars represent Standard error of the mean that was calculated from three consecutive exposures to the same concentration.

Figure 6 and Figure 7 illustrate the response/recovery time dependences on the WE surface area for different analyte flow rates (Figure 6a and Figure 7a) and on the analyte flow rates for different WE surface areas (Figure 6b and Figure 7b). It can be reasonably said that the influence of WE surface area on both the response/recovery time was negligible. Differences from all measurements fell within the range of ±2 s without any clear trend with respect to the WE surface area. This fact is not surprising because the area of the working electrode, which is closely related with the area of the electrochemical active interface between the SPE layer and the working electrode, usually influence the sensor sensitivity. Response/recovery time of the sensor is usually influenced by the transport of the analyte to the electrochemically active (electrode-electrolyte) interface. Within our experiments, sensors were not intentionally protected any diffusion barrier that usually controls the transport of the analyte through the sensor housing and so the only thickness of the working electrode, through which the analyte penetrated to the interface with the SPE, could significantly influence response/recovery time. Although we do not have exact values of WE thicknesses for particular sensors with different WE areas we suppose that particular working electrodes were nearly the same thickness which resulted in the negligible effect of the WE surface area on the response/recovery time. Figure 6b and Figure 7b show response and recovery time dependence on the analyte flow rate. While a slight increase in both response/recovery time was observed when the analyte flow rate was decreased from 1 L/min to 0.5 L/min, a substantial increase in both times was observed when the analyte flow rate was set to 0.1 L/min. However, this fact could have partially contributed to the time period which is necessary to exchange the internal dead volume of the test chamber (90 mL).

**Figure 8:**
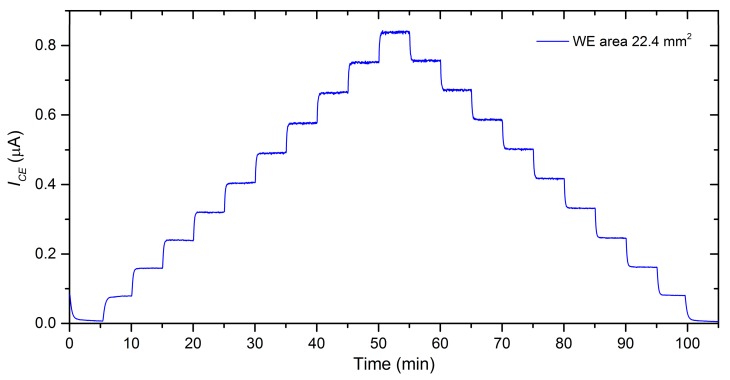
Sensor response to stepwise increase in NO_2_ concentrations within the range of 0−1 ppm (1 step equaled 100 ppb), WE surface area of 22.4 mm^2^ conditions: 298 K, 40 %RH, analyte flow rate 1 L/min.

The LOD calculated as the ratio of the triple standard deviation of the background current noise (at zero concentration) and sensitivity is a rather theoretical value that can usually be verified with difficulty. First, an accurate and reproducible preparation of such low concentration levels (ideally equaling the calculated detection limits) is very problematic. In our case, the testing apparatus allowed a minimum step increase equaling 100 ppb NO_2,_ which resulted in an average increase of 83 nA of the reduction current I_CE_ within the range of 0−1ppm NO_2_ (see Figure 8).

**Figure 9:**
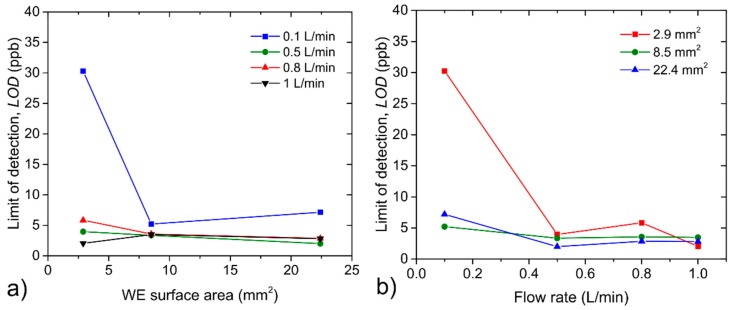
(**a**) Limit of detection (LOD) dependence on the WE surface area, (**b**) LOD dependence on the analyte flow rate.

Thus, if the sensor was exposed to the calculated detection limit (units of ppb for sensor with the WE surface area of 22.4 mm^2^, see Figure 9) an expected current response would be within the range of units of nanoampere (nA). Although such a low current response can be measured, there is no guarantee whether such a small change in the measured current cannot be attributed to some parasitic events. Second, the activation of the electrode surface may require a specific amount of analyte and so the calculated detection limit may naturally differ from the actual value. Nevertheless, LOD is indisputably a sensor parameter that contributes to the determination of the overall sensor performance and is worth determining; especially when we are not primarily interested in the absolute value of LOD but rather in its potential dependence on WE surface area and/or analyte flow rate (see Figure 9). While the WE surface area had a minor impact on LOD for analyte flow rates of 0.5, 0.8 and 1 L/min, a substantial increase of the LOD value was observed for the smallest WE area (2.9 mm^2^) at the lowest analyte flow rate (0.1 L/min). This fact was probably caused by a combination of low sensitivity and non-ideal (non-constant) background current, which resulted in a higher value of the background current noise. A similar result can be seen in Figure 9b, where negligible LOD dependence on analyte flow rate was observed, with the exception of the sensor with the smallest WE surface area at the lowest analyte flow rate.

**Figure 10:**
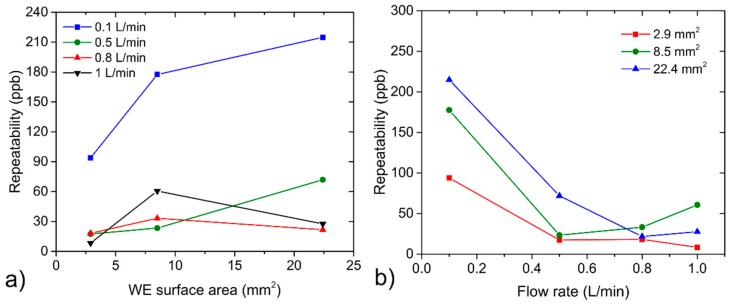
(**a**) Repeatability dependence on the WE surface area, (**b**) repeatability dependence on the analyte flow rate.

Repeatability may provide information about the short-term stability of sensor response. This parameter was determined as a ratio of the triple standard deviation of three consecutive measurements (see Figure 4) when the tested sensor was exposed to the same concentration level and sensitivity. Figure 10a,b show repeatability dependences on the WE surface area and the analyte flow rate. The measurement of sensor repeatability was naturally influenced by the repeatability of the actual concentration level in the test chamber itself. Because the actual concentration level at repeated exposures could fluctuate within the range of tens of ppb (the worst value is ±40 ppb at flow rate 0.5 L/min, given by the repeatability of the particular flow through the mass flow controller), it is necessary to take this fact into account and discuss only clear general trends. Figure 10a indicates changes of repeatability without a clear trend in dependence on WE surface area for the flow rates of 0.5, 0.8 and 1 L/min, while considerable growing dependence was observed for the flow rate of 0.1 L/min, which resulted in worse repeatability for a larger WE surface area. Figure 10b shows relatively minor changes in sensor repeatability within the range of 0.5−1 L/min, while substantially worse values of repeatability were observed for all WE surface areas when analyte flow rate was decreased to 0.1 L/min. The response of the sensors showed a significant dependence on the mutual orientation of the sensor and the analyte flow direction, as well as on the location of the sensor in the test chamber. Considering previous measurements, further experiments regarding sensor orientation and location were focused only on the sensor with the largest working electrode.

### 3.2. Effect of the Sensor Rotation on Sensor Parameters

**Figure 11:**
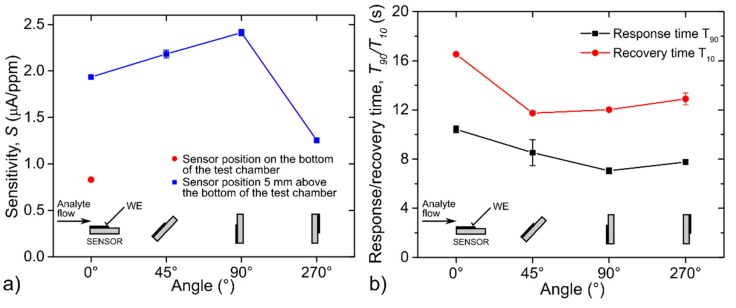
(**a**) Sensitivity dependence on the mutual orientation of the analyte flow direction and the WE surface area (22.4 mm^2^); (**b**) response/recovery time dependence on the mutual orientation of the analyte flow direction and the WE surface area (22.4 mm^2^); conditions: 298 K, 40 %RH, analyte flow rate 1 L/min. The error bars in Figure 11a represent the standard error of the slope of the calibration curve (sensitivity) that was constructed by linear regression. The error bars in Figure 11b represent standard error of the mean that was calculated from three consecutive exposures to the same concentration.

The second set of experiments was aimed at the sensor with the largest WE surface area, where attention was paid to the effect of the mutual orientation of the WE surface area and the analyte flow direction on sensor parameters. The sensor was connected via silver wires and placed approximately 5 mm above the chamber bottom (see Figure 3). We exposed the sensor to a test cycle (Figure 4) under four different mutual orientations of analyte flow direction and sensor surface (angles of 0°, 45°, 90° and 270°, see Figure 11). Sensor sensitivity gradually increased as the sensor was rotated from the 0° angle position (analyte flow direction was parallel to the WE surface area) to the 90° angle position (analyte flow direction was perpendicular to the WE surface area). The mutual angle of 270° (the sensor was facing away from the analyte flow direction) caused a rapid decrease in sensitivity. An interesting feature was observed when the sensitivity of the sensor placed at the chamber bottom (red point in Figure 11a, mutual angle near 0° is expected) was compared with the results of the rotated sensor from Figure 11a. It was observed that the sensor position in *z* axis, i.e., the height of the sensor above the chamber bottom, was important and although both sensitivities for these positions could be expected to be similar, they differed markedly. Considering these facts, it is obvious that the number of detected gas molecules, which hit directly the surface of WE, per second increases with the angle (from 0° up to 90°), the current via sensor grows (described in detail in the Section 3.3.) and the sensitivity is higher (see Figure 11). Thus, we could assume that the sensitivity is related to the to the pressure exerted on sensor, i.e., to fluidic conditions around the sensor. Response/recovery time dependence is shown in Figure 11b. Generally, response times were lower than recovery times for all mutual orientation angles between analyte flow and the WE surface area. Both the response/recovery time showed a slightly decreasing trend as the mutual angle was increased. When the sensor was facing away from the analyte flow (270° angle) both times increased slightly. The only exception was observed with respect to the aforementioned description. The change of mutual angle from 45° to 90° resulted in minor increase of the value of recovery time in contrast to the continual decreasing trend of response time. This fact does not have a clear explanation and it could be caused by specific fluidic conditions around the sensor or attributed to a not quite stable current response of the sensor at the angle of 45°.

**Figure 12:**
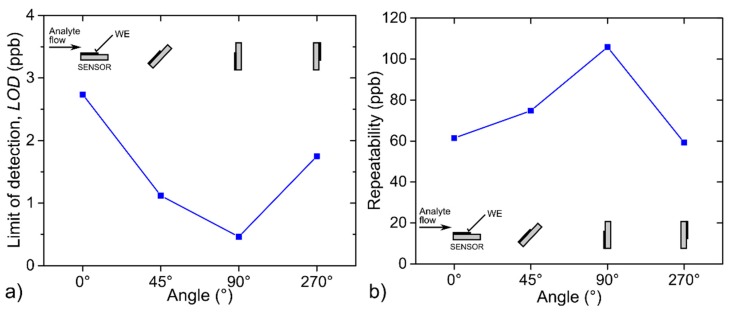
(**a**) LOD dependence on the mutual orientation of analyte flow direction and the WE surface area (22.4 mm^2^); (**b**) repeatability dependence on mutual orientation of analyte flow direction and WE surface area (22.4 mm^2^); conditions: 298 K, 40%RH, analyte flow rate 1 L/min.

The impact of sensor rotation on the limit of detection and the repeatability of sensor response is shown in Figure 12. LOD decreased as the angle of rotation increased, except the angle value of 270°. Because LOD was calculated as the ratio of triple standard deviation of background current noise and sensitivity, it can be reasonably expected that the LOD dependence on the rotation angle would follow exactly the opposite trend to sensitivity dependence in Figure 11. Repeatability was deteriorating with the increasing angle of rotation from 0° to 90°. When the angle of 270° was set, the value of repeatability decreased to almost the origin value at the 0° angle.

### 3.3. The Effect of the Sensor Orientation on Direct Current (DC) Response and Current Fluctuations under Equilibrium Conditions 

The signal responses of the largest working electrode were further investigated under equilibrium conditions when the sensor was being kept at particular conditions (concentration and flow rate) for the required amount of time to fulfill the memorylessness of current fluctuations. These measurements of DC current and its fluctuation were also carried out (i) for a range of concentration at the constant flow rate (1 L/min) and (ii) for a range of flow rates at a constant concentration (NO_2_ 3 ppm). All measurements under equilibrium conditions were carried out as follows. First, a particular NO_2_ concentration (e.g., 1 ppm) was set with the particular total flow rate (e.g., 1 L/min) in the conditions of 298 K, 40%RH. The DC current via a sensor was measured and monitored until it did not change its mean value for 100 seconds. After this (approximately 300 s from the beginning of the procedure), we ran the current fluctuation measurement with a DC current measurement at the same time to further estimate the signal-to-noise ratio. 

**Figure 13:**
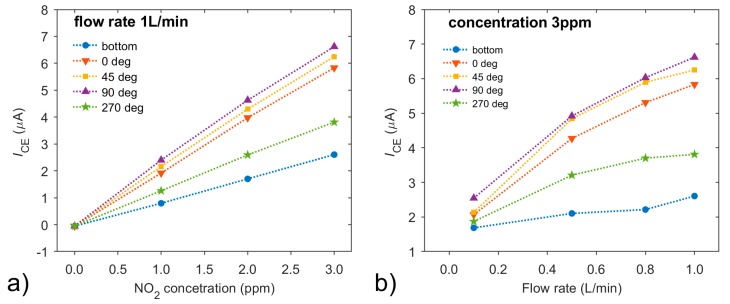
Dependences of direct current (DC) (**a**) on NO_2_ concentration at the constant flow rate of 1 L/min; or (**b**) on the flow rate at the constant concentration of 3 ppm, with sensor orientations of 0°, 45°, 90° and 270°. Conditions: 298 K, 40 %RH.

Figure 13a shows the dependences of DC current on concentration at the constant flow rate of 1 L/min, where the highest DC response corresponds to a sensor orientation in which a flow of analyte incidents perpendicularly on to the working electrode (90°). As concentration increases, the DC component linearly increases for all orientations. Figure 13b illustrates how the DC current varies with the flow rate and sensor orientation at the constant NO_2_ concentration of 3 ppm. The DC current increases with the flow rate as described in our previous paper [21], The current varies even at the lowest flow rate and its dependence develops as expected; the larger the area of the working electrode directly affected by the incident analyte flow as the sensor is rotated, the higher the current through the sensor. The sensor rotated by 270° or the sensor located at the bottom of the gas chamber exhibit the lowest DC response to the change of flow rate as well as to the change of NO_2_ concentration. Thus, we might assume that the sensor response is a result of fluidic conditions influenced by geometry, location and orientation of the sensor itself. This assumption is further elaborated in the next section, where the measurement of current fluctuations is discussed.

#### 3.3.1. The Effect on Current Fluctuation 

**Figure 14:**
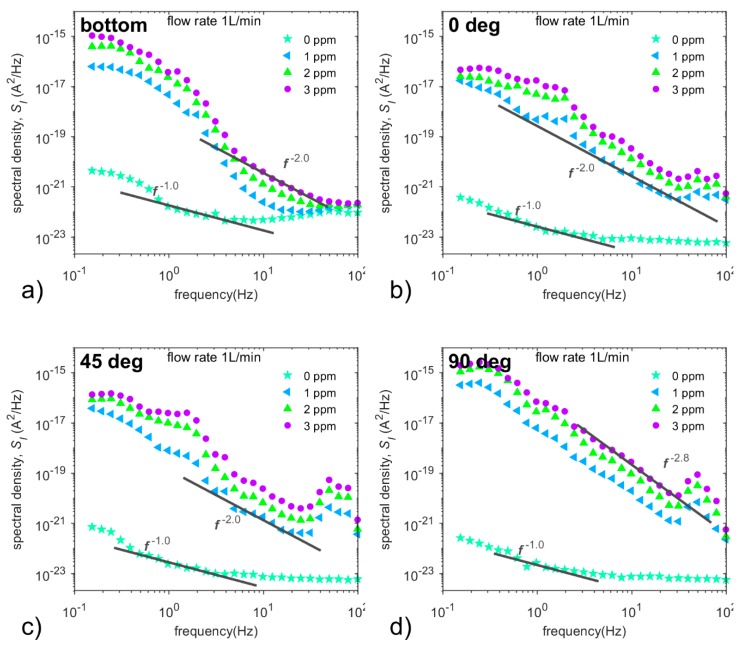

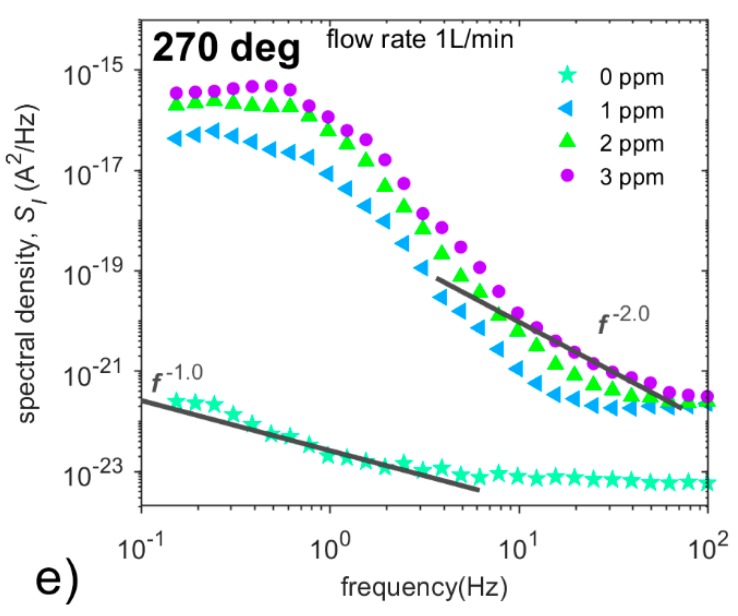
Spectral densities of current fluctuations depending on NO_2_ concentrations at the flow rate of 1 L/min for the frequency range from 0.1 Hz up to 100 Hz for the sensor located: (**a**) at the bottom of the gas chamber, and for the sensor located in the middle of the gas chamber at the angles of (**b**) 0°, (**c**) 45°, (**d**) 90° and (**e**) 270°. Conditions: 298 K, 40%RH.

Figure 14 presents spectral densities of current fluctuations depending on NO_2_ concentrations at the flow rate of 1 L/min for the frequency range from 0.1 Hz up to 100 Hz as the sensor location and sensor orientations are changed. At zero concentration, power spectra exhibit a significant thermal noise, associated with all dissipative processes across the sensor and 1/*f* noise component; this noise indicates diffusion-dominant electrode electrolyte interface [21,30,31,32]. We need to point out that the sensor located in the air (0°, 45°, 90° and 270°) exhibits a different shape of thermal noise in comparison to the sensor located at the bottom of the chamber. The cause is assumed to be due to the different overall capacity of the sensor, which is influenced by the relative permittivity of material under the ceramic substrate (air vs. Polytetrafluoroethylene).

When the concentration rises to 1 ppm of NO_2_, the spectral densities of current fluctuations contain (i) the noise component *f*^−2^, indicating adsorption-desorption noise or drift-dominant electrode/electrolyte interface [30], (ii) noise component of Lorentzian-a-like spectra assumed to be the result of several mutually influencing stochastic processes [21], (iii) a wide peak at frequencies from 45 Hz up to 60 Hz and (iv) thermal noise, which increases due to a change of sensor capacity. The peak is noticeable only in the orientations of 0°, 45° and 90°, where its position, width and size change as the analyte flow directly impacts the active layer of the sensor at different angles. The additional increase of NO_2_ concentration leads to a shift of power spectra to the higher values, but their shape remains unchanged. Thus, the shape of current noise spectral densities develops according to the orientation of the sensor towards the incident analyte flow and sensor position.

**Figure 15:**
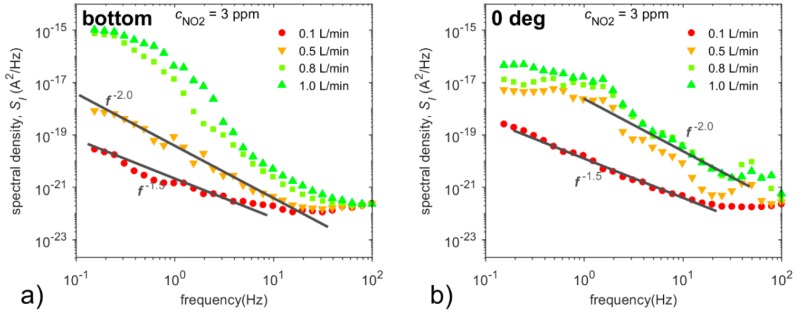

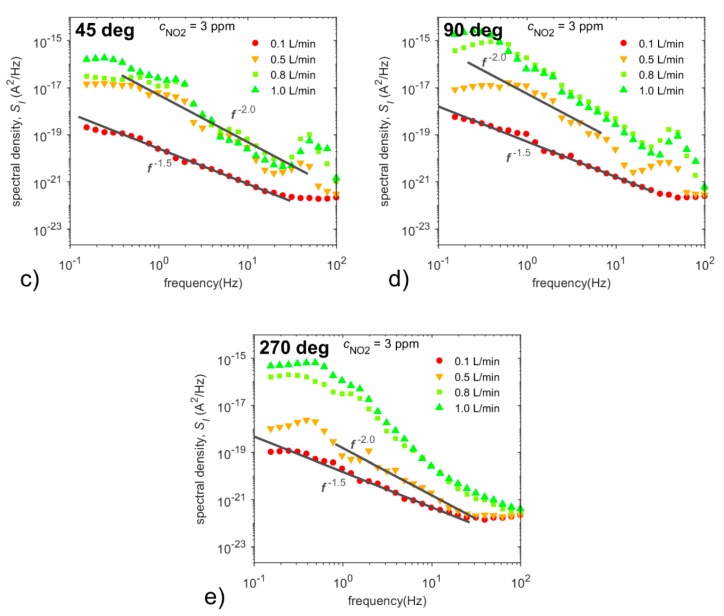
Spectral densities of current fluctuations depending on flow rates at NO_2_ concentrations of 3 ppm for the frequency range from 0.1 Hz up to 100 Hz for the sensor located: (**a**) at the bottom of the gas chamber, and for the sensor located in the middle of the gas chamber at the angles (**b**) 0°, (**c**) 45°, (**d**) 90° and (**e**) 270°. Conditions: 298 K, 40 %RH.

To confirm this assumption, the dependence of spectral densities on the flow rate has to be investigated. Figure 15 shows spectral densities of current fluctuations depending on flow rates at NO_2_ concentration of 3 ppm for all sensor locations and sensor orientations. At the low flow rate of 0.1 L/min, thermal noise and diffusion noise (*f* ^−1.5^) dominate in spectral densities of current fluctuations in all of the sensor orientations and positions. The power spectra exhibit the same shape but not the same level; see Figure 15a,d. The same shape of the power spectrum together with different DC responses (see Figure 13b) for particular cases indicate that current fluctuations are not influenced by flow around the sensor at this flow rate. 

Figure 15 further demonstrates that the spectral densities of current fluctuations contain significant *f*^−2^ noise, especially at the flow rate of 0.5 L/min, while the Lorentzian-a-like noise component and the wide peak in spectra prevail at the flow rates of 0.8 L/min and 1 L/min. These two components are assumed to be the result of several mutually influencing stochastic processes [21], which could include the noise arising from the flow around the sensor (partial pressure fluctuations) [33], velocity fluctuation due to turbulent flow [34], diffusion noise, adsorption-desorption noise and 1/f noise. 

By comparing current noise spectra at increasing flow rates ranging from 0.5 L/min to 1.0 L/min, one can observe how the flow around the sensor affects these spectral densities in a specific way for each orientation and position of the sensor with one exception: the noise spectra for the sensor located at the chamber bottom and the sensor located in the air with a 270° orientation show a certain degree of similarity. It is worth noting that the sensor is not directly affected by the analyte flow in these positions, unlike in other positions, as mentioned above. Furthermore, we suppose that the effect of turbulent flow on current fluctuations via the sensor is the most significant in these two positions as Lorentzian-a-like component (caused by velocity fluctuation [34]) develops with increasing degree of turbulence (see Figure 15a,e) in the frequency range below 2 Hz. Thus, current noise is affected not only by the dimensions of the test chamber [21], but mainly by the flow around the sensor (i.e., construction of the sensor, etc.), as shown in Figure 14 and Figure 15.

#### 3.3.2. The Effect on Signal-to-Noise Ratio 

To summarize and evaluate the results, the relation between DC current and current fluctuations is introduced as the signal-to-noise ratio for the selected frequency range (SNR_Δf_):

(1) $$SNR_{\Delta f}=20\log\frac{I_{DC}}{\sigma_{\Delta f}}\;\left[dB\right],$$

where *I*_DC_ represents the DC current and $\sigma_{\Delta f}$ denotes the standard deviation, which equals the square root of the integral of the spectral density over the defined frequency range for a particular concentration and flow rate:

(2) $$\sigma_{\Delta f}=\sqrt{\int_{f_{1}}^{f_{2}}S_{I}\left(f\right)df},$$

where limits *f*_1_ and *f*_2_ define the frequency range. In our case, the frequency range was from 0.1 Hz to 100 Hz. Sensor orientation, in which a flow of analyte incidents directly and perpendicularly onto the working electrode (90°), exhibits the highest DC component 2.4μA/ppm and also the highest level of fluctuations at the flow rate of 1 L/min, but low signal-to-noise ratio. 

**Figure 16:**
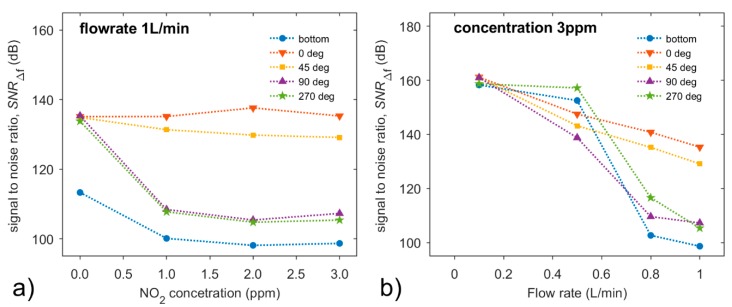
Dependences of signal-to-noise ratio (**a**) on NO_2_ concentration at the constant flow rate of 1 L/min, or (**b**) on the flow rate at the constant concentration of 3 ppm, at the orientations of sensor - 0°, 45°, 90° and 270°. Conditions: 298 K, 40%RH.

Figure 16a shows that *SNR*_Δ*f*_ remains almost at the same level for non-zero concentration while it depends significantly on the orientation and position of the sensor according to incident analyte flow of the non-zero flow rates. Surprisingly, the highest *SNR*_Δ*f*_ is exhibited by the position where the sensor was placed in the air with a 0° rotation. Figure 16b shows that the value of *SNR*_Δ*f*_ decreases as the flow rate increases as published before [21]. Even though all dependences are non-ascending curves, their shapes differ as the rotation of the sensor changes.

## 4. Conclusions

The paper presents an experimental study of how the current via amperometric sensor as well as the spectral density of its fluctuations develop in relation to the flow rate, the concentration of the detected gas and the orientation of the sensor towards the incident analyte flow in the gas chamber. The raw data from all measurements are available online at [35] Mendeley Data.

Sensor performance represented by selected sensor parameters was influenced by both the analyte flow rate and the orientation of the sensor towards the analyte flow direction. Sensitivity showed a linearly increasing trend depending on the analyte flow rate, and the trend was gradually steeper as the WE surface area increased. While the response/recovery time naturally increased with decreasing analyte flow rates, the effect of the size of the WE surface area to both times was negligible. The limit of detection was almost independent of both the size of the WE surface area and the analyte flow rate with a single exception. The combination of the lowest flow rate together with the smallest WE surface area caused a substantial increase of the LOD value. The repeatability of sensor response did not show any clear trend with respect to the size of the WE surface area, except for the sensor with the smallest WE surface area. The analyte flow rate had a clearly negative impact on repeatability only for the lowest value tested, i.e., 0.1 L/min. The effect of sensor rotation on selected parameters was studied only for the largest WE surface area. All sensor parameters except repeatability (i.e., sensitivity, response/recovery time and LOD) were improving with the increasing angle, from 0° (analyte flow direction was parallel to the WE surface area) to 90° (analyte flow direction was perpendicular to the WE surface area). When the sensor was facing away from the analyte flow direction (angle 270°), all sensor parameters, except repeatability, were slightly worse.

At the very low flow rate of 0.1 L/min, the current fluctuations and the DC component show minimum dependence on the orientation of the sensor towards incident analyte flow. As the flow rate increases at the constant concentration of the detected gas, the DC component of the current through the sensor shows non-linear increments within a range below an order, while the spectral density of current fluctuations significantly changes its shape and level of current fluctuations in the power spectrum for the orders. It needs to be pointed out that current noise spectra at increasing flow rates from 0.5 L/min to 1.0 L/min develop in a specific way for each orientation and position of the sensor with an exception—the noise spectra for the sensor located at the chamber bottom and the sensor located in the air with a 270° orientation show a certain degree of similarity. 

Considering the constant flow rate (1 L/min), the increase of NO_2_ concentration leads to a shift of the power spectra towards higher values, but their shape remains unchanged for a particular sensor orientation and position. Thus, the current noise is affected not only by the dimensions of the test chamber, but mainly by the flow around the sensor since the fluidic environment in the vicinity of the sensor´s active layer is the function of the fluidic conditions in the chamber determined by the flow rate, sensor location and sensor orientation.

The signal-to-noise ratio, *SNR*_Δ*f*_, depends on the flow rate and sensor orientation to incident flow, but it is almost invariant to various concentrations of the detected gas. The value of *SNR*_Δ*f*_ decreases as the flow rate increases; however, all dependences are not non-ascending curves and their shapes differ as the sensor rotation changes.
